# Navigating Antimicrobials and Combating Antimicrobial Resistance: Challenges, Impacts, and Strategies for Global Action

**DOI:** 10.7759/cureus.82064

**Published:** 2025-04-11

**Authors:** Vishnu Desai, Santosh Kumar, Bhavin Patel, Shirishkumar N Patel, Hiren H Patadiya, Deeksha Asawa, Mohd. Shabankhan H Pathan, Mainul Haque

**Affiliations:** 1 Department of Periodontology and Implantology, Karnavati School of Dentistry, Karnavati University, Gandhinagar, IND; 2 Department of General Dentistry, My Dental Southbridge PLLC, Southbridge, USA; 3 Department of Pharmacology and Therapeutics, National Defence University of Malaysia, Kuala Lumpur, MYS; 4 Department of Research, Karnavati School of Dentistry, Karnavati University, Gandhinagar, IND

**Keywords:** antibiotic misuse, antibiotic overuse, antimicrobial stewardship programs (asps), drug resistance, global health, infection control, infectious diseases, one health approach, public health, resistance to antimicrobial drugs

## Abstract

Antimicrobial resistance (AMR) is one of the biggest problems facing the scientific and medical communities. According to WHO, this growing issue might make once-effective antibiotics obsolete and pose a substantial risk to public health. Estimates indicate that multimillion deaths were either directly or indirectly caused by AMR, making it one of the most substantial risks to public health and development in the world. The issue of AMR is primarily caused by healthcare workers’ excessive and inappropriate use of antimicrobial agents. Dentists are believed to prescribe a considerable portion of all antibiotics globally. The emergence of AMR, its causes, and its effects on human health are examined in this article, with special attention to dental offices and medical facilities. It draws attention to the rising issue of antibiotic overprescription and abuse, particularly in low- and middle-income countries, where improper antibiotic use is an everyday practice around the globe. The article discusses the role of antimicrobial stewardship programs and the importance of implementing precise, evidence-based practices in preventing AMR. Since antibiotic abuse in livestock greatly accelerates the spread of resistance, the role of antibiotics in animal agriculture is also investigated. To address AMR, the paper highlights the necessity of a global, coordinated response that bolsters surveillance systems, cuts back on needless antibiotic use, and expands access to alternative treatments. Recent research has called into question the efficacy of preventive antibiotic medication in these situations. According to other researchers, it might not help avoid surgical site infections. However, other experts say disrupting deeper tissues and local mucosal defenses during an intraoral surgical operation may raise the risk of infection even when antibiotics are used.

## Introduction and background

Sir Alexander Fleming invented penicillin in 1928, and in the latter half of the 20th century, significant advancements in the research and development of anti-infection medications were made, resulting in more effective treatments and better control of infectious diseases [1]. However, the overuse and misuse of antibiotics in animal feed have caused antimicrobial resistance (AMR) to rise rapidly. Multidrug resistance (MDR) has become a significant public health and economic issue affecting both the livestock sector and human healthcare, leading to the failure of traditional medical treatments [2].

AMR is one of the most critical problems facing the scientific and medical communities [3,4]. AMR is a serious public health concern that can render once-effective medications ineffective, according to WHO [5]. Resulting in an estimated 4.95 million deaths either directly or indirectly associated with AMR, it is regarded as one of the most considerable risks to global public health and development [6].

One of the leading causes of this problem is the excessive or inappropriate use of antimicrobial agents by medical personnel. A total of 10-13% of all antibiotic prescriptions worldwide are thought to be for dental purposes [7]. From essential extractions of residual roots and periodontal and endodontic surgeries to more involved and invasive procedures that frequently involve multidisciplinary expert teams in a hospital setting, dentists in various specializations often perform dental surgery. Because they usually entail manipulation of the gingival and periapical regions, with or without mucosal perforation, these procedures are similar in that they are invasive [8].

According to recent research, prophylactic antibiotic therapy may not always be necessary in certain situations. It might not help prevent postoperative infections, according to some researchers. However, according to other experts, the mucosa’s and deeper tissues’ local defenses are weakened once an intraoral surgical operation starts, which could raise the danger of infection [9].

The World Dental Federation’s 2019 General Assembly covered practical strategies for dentists to help WHO’s 2015 worldwide drive against bacterial resistance, which aims to reduce the overprescription of antibiotics [6]. In some situations, using antibiotics for just one day can encourage the development of a resistant disease. Customized medications made possible by personalized healthcare practices can enhance health outcomes and delay the emergence of resistance [6]. Experts advise many tactics to lessen the effects of bacterial resistance, including making a precise diagnosis based on scientific data; choosing an antibiotic with a narrow spectrum, if at all possible; using the proper dosage for the type and site of infection; assessing the patient 48 hours after beginning antibiotic therapy to avoid needlessly finishing a course of antibiotics without showing clinical improvement; and increasing understanding of various antimicrobial classes and treatment procedures to prevent indiscriminate use [6,10].

Problem statement

A considerable health threat to global health, AMR makes once-effective antibiotics useless and raises mortality and medical expenses. Resistance is accelerated by the overuse and abuse of antibiotics in dentistry, medicine, and agriculture, particularly in low- and middle-income (LMIC) nations with lax laws. AMR makes treating infections more difficult, which puts further strain on healthcare systems. Multidrug-resistant diseases are still rising despite international efforts, such as WHO initiatives and antimicrobial stewardship programs (ASPs). Stricter antibiotic laws, better surveillance, and increased public awareness are all necessary to combat AMR, encourage responsible antibiotic use, and avert a time when common diseases will no longer be treatable.

Objectives

This article outlines the causes, effects, and worldwide implications of AMR. It highlights the difficulties in preventing AMR, especially in LMICs, by examining the impact of antibiotic abuse and overuse in dentistry, medical, and agricultural settings. The study assesses how well ASPs work to cut down on needless antibiotic use. It suggests international solutions like enhanced surveillance, more stringent laws, and public awareness campaigns. Finally, it highlights the necessity of a concerted global response to stop the spread of illnesses resistant to drugs and protect public health.

## Review

Materials and methods

**Figure 1:**
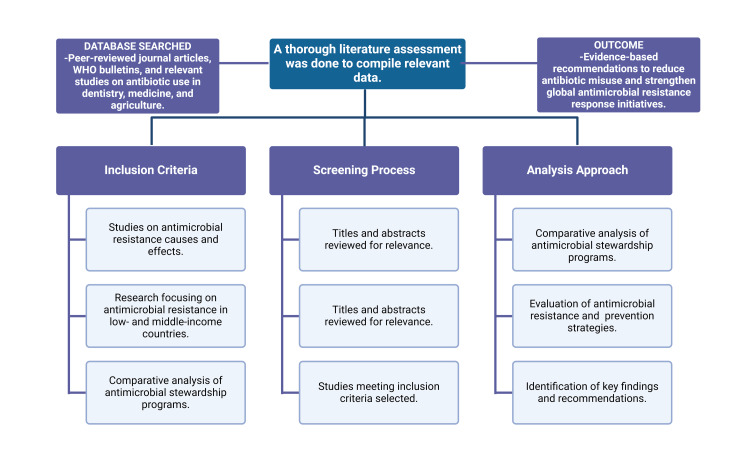
Flowchart illustrating the methodology of the study This figure was created using the premium version of BioRender (biorender.com) under agreement license number OR281OVKBP. Illustration credit: Vishnu Desai

A thorough analysis of the body of research, studies, and international guidelines on AMR served as the foundation for this essay. The data sources were peer-reviewed journal papers, WHO bulletins, and pertinent studies on antibiotics in dentistry, medical, and agricultural settings. The project’s primary goal is to find the origins, effects, and tactics for preventing AMR, especially in LMICs. It also uses comparative analysis to assess the efficacy of ASPs. The results are combined to provide evidence-based suggestions for lowering antibiotic abuse and bolstering international AMR response initiatives. The methodology of this narrative review is illustrated in Figure 1.

Review of literature

Overuse of Antibiotic Prescriptions

The World Dental Federation reviewed practical ways for dentists to support WHO’s 2015 global action plan against bacterial resistance during its 2019 General Assembly [11]. The goal was to reduce the overprescription of antibiotics and ensure that dentists are essential to the fight against AMR [6,11]. According to reports, dentists prescribe antibiotics in about 10% of cases, with 66% of these prescriptions having no therapeutic justification. This highlights the need for improved surveillance of antibiotics in the dental profession to reduce unnecessary prescriptions and fight AMR [12]. Mainly in the following situations are prescription antibiotics advised in dentistry: (a) to treat an existing infection in combination with other treatments; (b) to prevent the infection from propagating; (c) when there is systematic involvement; and (d) if the patient is vulnerable to complications and clinically compromised. Following these recommendations, antibiotics are used sensibly and only when required [13].

Resistance to Antimicrobial Therapy

**Figure 2:**
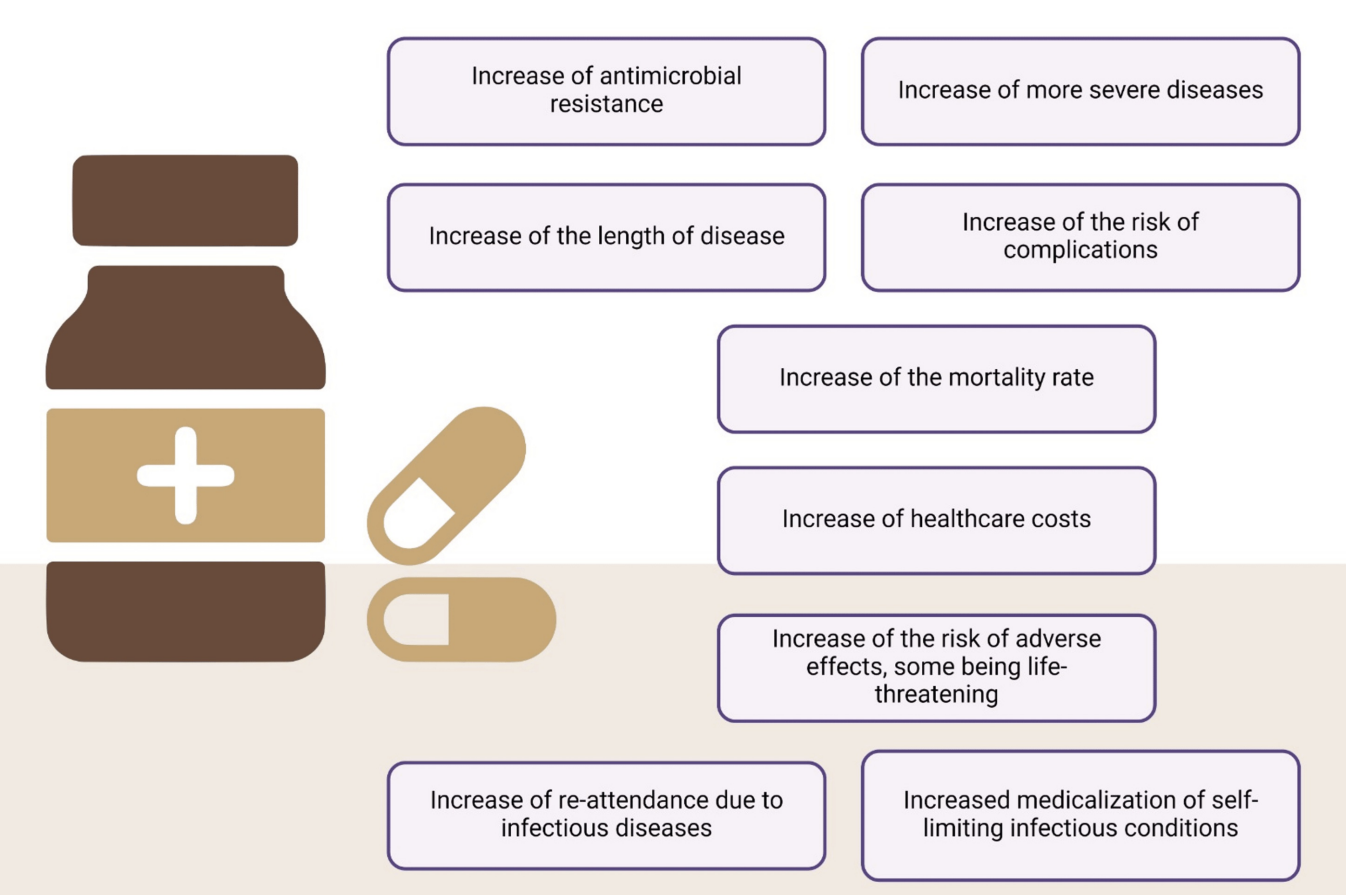
Risks associated with antibiotic overuse This figure was created using the premium version of BioRender (biorender.com) under agreement license number FH280EPUMH. Illustration credit: Vishnu Desai

It is commonly acknowledged that one of the most significant risks to human health in the world is AMR. For instance, methicillin-resistant *Staphylococcus aureus *simply causes more deaths in the United States than emphysema, HIV/AIDS, Parkinson’s disease, and homicide altogether. This harsh reality emphasizes how urgently improved antibiotic stewardship and preventative measures are needed to combat AMR [14]. Strains resistant to isoniazid and rifampicin are thought to be responsible for 20% of previously treated cases and 3.7% of newly diagnosed cases of tuberculosis (TB) worldwide. Despite decades of success, these two anti-TB medications are no longer effective enough. The fact that roughly half of MDR TB infections are currently successfully treated with the drugs already on the market emphasizes the increasing difficulty of treating TB due to drug resistance [14]. MDR-TB plus resistance to either fluoroquinolone or any second-line injectable drug is known as extensively medication-resistant TB, and it has been found in 84 countries worldwide. This makes treating TB much more complicated and emphasizes how urgently new treatment alternatives and more effective strategies to counteract drug resistance are needed [14]. In primary care, AMR is associated with TB, gonorrhea (particularly *Neisseria gonorrhoeae*), typhoid fever, and Group B streptococcus. AMR is becoming a more significant public health concern as these diseases are getting harder to treat because they are resistant to standard medications [14]. AMR microbial infections can cause severe sickness, increased mortality, and a higher risk of complications, which frequently call for hospitalization (Figure 2). Managing such illnesses becomes more complicated when resistance reduces the efficacy of conventional therapies, further taxing healthcare resources [14]. The European Centre for Disease Prevention and Control reports that antibiotic resistance claims the lives of 35,000 people in Europe annually [15].

Furthermore, antibiotic resistance substantially increases healthcare expenses, with related issues costing Europe an estimated €11.7 billion annually [16]. A recent analysis estimated that antibiotic resistance could add up to £20,000 in hospital costs per patient episode [14]. Most antibiotics are prescribed by general practitioners (GPs), with primary care physicians responsible for 80-90% of all antibiotic prescriptions in Europe. Respiratory tract infections are the leading cause of antibiotic prescriptions. Antibiotics are widely used in other sectors, particularly farming, aquaculture, and agriculture, collectively accounting for approximately 80% of antibiotic consumption in the United States [14].

Southern and Eastern European countries utilize antibiotics at far higher rates. In Turkey, it was reported that 42.3 defined daily doses (DDDs) were used [17,18]. Antibiotics are responsible for approximately 20% of all related drug ED visits in the United States, with allergic reactions forming most of these visits [19]. Nonetheless, several frequently given antibiotics have been connected to side effects that range from neurological and behavioral conditions to gastrointestinal issues. Although the majority of these side effects are minor, there have occasionally been reports of severe, potentially fatal disorders, including hepatotoxicity with amoxicillin and clavulanate [14].

Making Necessary Antibiotics Accessible, Available, and Used Appropriately

AMR, which is causing increased illness, death, and healthcare expenses, is still a primary global concern, especially in LMICs [20,21]. Although AMR is present everywhere, LMICs have a disproportionately higher incidence of the disease, a condition made worse by the COVID-19 pandemic and other current issues [22,23]. Currently, an estimated 80% of the roughly 10 million fatalities worldwide are attributed to AMR occurring in LMICs [24,25]. In LMICs, the persistently high rates of inappropriate antibiotic prescribing and the self-medication of antimicrobials and defective dispensing of antibiotics, often through informal sellers, for minor or self-limiting infections, like upper respiratory tract infections (URTIs), are essential factors that contribute to the exacerbation of AMR [23,26-28]. This is particularly important since primary care, which includes hospital outpatients, accounts for around 90% of all antibiotic use in humans in LMICs [29]. AMR is also exacerbated by the prevalence of counterfeit and subpar antibiotics in LMICs [30]. Essential antibiotics are consistently available in healthcare settings, including basic healthcare facilities; however, this is impacted by supply chain issues and medication shortages in LMICs. AMR may increase [31,32]. It can be difficult to guarantee adequate access to antibiotics in many LMICs, which could raise mortality, raise healthcare expenses, and aid in the spread of AMR [33,34]. WHO’s Access, Watch, and Reserve (AWaRe) classification system includes the “Access” antibiotic category. These antibiotics are advised for usage as first-line therapies in medical settings and are thought to be necessary for treating common illnesses [35,36]. Affordability concerns can exacerbate access to and proper use of antibiotics in many Asian and African nations, especially when there are significant patient co-payments. This is demonstrated when patients cannot purchase many antibiotic pills or use leftover medicines, such as those kept at home or shared by family and friends, before consulting a doctor or dispenser, which puts further financial hardship on them [37]. The overprescription of antibiotics for self-limiting illnesses such as common colds, coughs, undifferentiated febrile fever, and other URTIs is a foremost concern for prescribers [38,39]. Since many people expect medicines to treat viral infections, including URTIs and severe diarrhea, patient pressure is probably a factor in inappropriate antibiotic prescribing in LMICs. Prescribers’ inadequate knowledge of antibiotics and AMR exacerbates this problem and leads to inappropriate prescribing practices [40,41]. Thankfully, in recent years, ASPs have proliferated in LMICs across all industries. Despite early worries about a lack of staff and resources, this expansion is meant to improve antibiotic use in the future [42,43]. Organizations, including WHO, the Fleming Fund, the British Society of Antimicrobial Chemotherapy, the Open University, and others, have backed the expansion of ASPs, which are essential in tackling antibiotic shortages. These organizations are crucial because they strengthen efforts to promote antimicrobial stewardship by offering top-notch training and improving connections across LMICs [44].

Antibiotic Therapy in Dentistry

Odontogenic and nonodontogenic infections are the main categories of orofacial infections. Odontogenic infections are diseases that start inside the tooth and its supporting tissues. However, nonodontogenic infections do not involve tooth structures [43-45]. Dental infections brought on by dental caries, pulpal necrosis, dental trauma, and periodontal disorders may have serious repercussions that impact the oral cavity’s soft and hard structures. Approximately 65% of orofacial infections are caused by Gram-positive cocci, while 25% of patient oral specimens include Gram-negative bacilli, per a prior study [45]. Pain and swelling in the mouth cavity are common signs of dental infections. Since these infections can cause serious and irreversible side effects such as osteomyelitis, brain abscess, airway blockage, carotid infection, sinusitis, septicemia, meningitis, cavernous sinus thrombosis, orbital abscess, and vision loss, they should be treated as soon as possible [46]. Antibiotic medications, endodontic therapy, and surgical procedures could all be used to treat dental infections [47]. Approximately 12% of dentists prescribe antibiotics as a preventative measure and treatment, according to earlier research [48]. Prescription antibiotics can have adverse side effects, including allergies, dermatological conditions, and hypersensitivity reactions [49]. 

**Figure 3:**
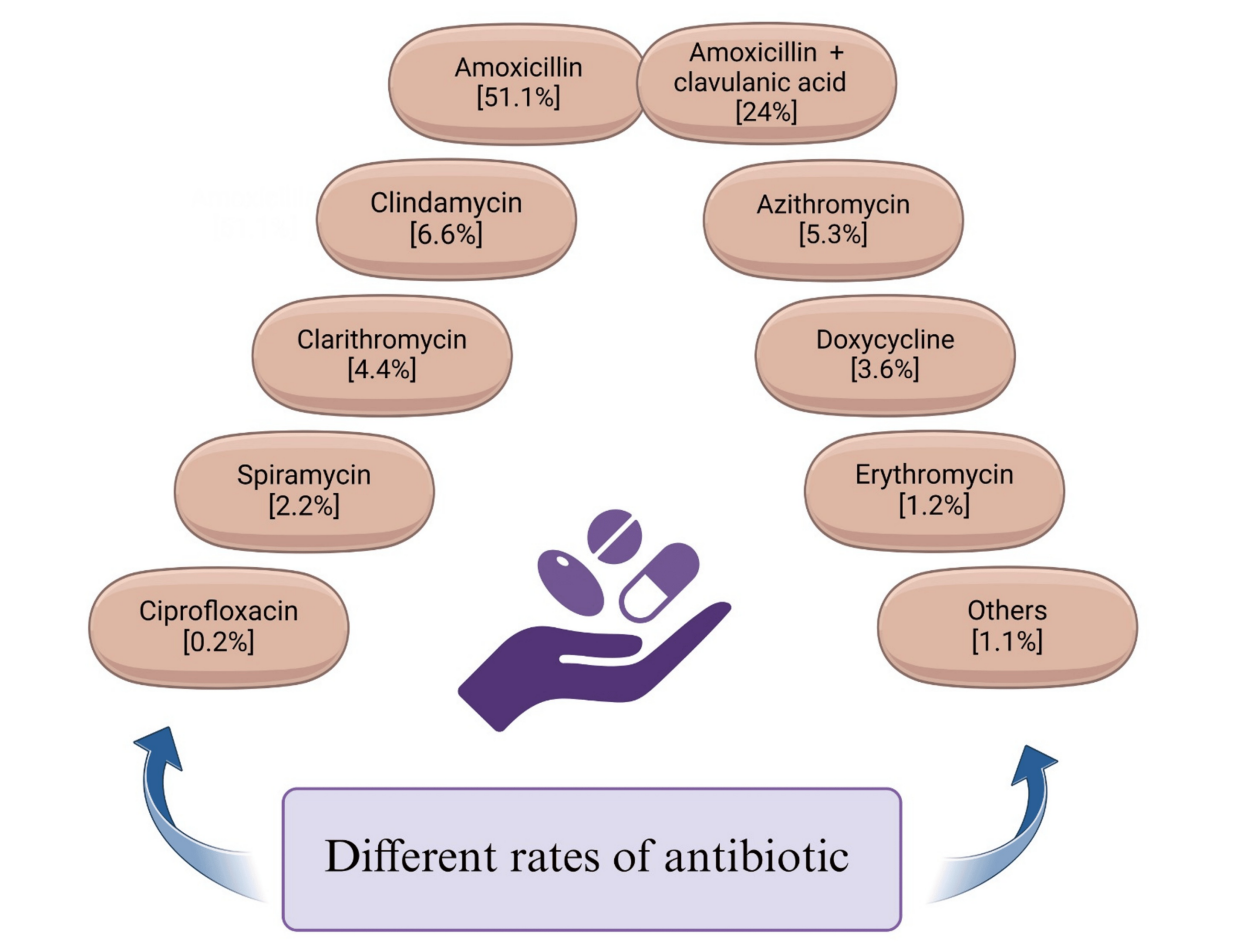
Different rates of antibiotic prescription by dentists This figure was created using the premium version of BioRender (biorender.com) under agreement license number LN281788N3. Illustration credit: Vishnu Desai

Additionally, overprescribing antibiotics may lead to many significant issues, such as bacterial resistance, hematological and gastrointestinal problems, and bacterial microbiota diversion [45,46]. This can result in oral bacterial resistance, a developing issue in medicine and dentistry. Antibiotics should only be used for acute infections and in a limited spectrum to avoid these issues. Additionally, more research and education must be done to stop and lessen the problem of antibiotic resistance [50]. Antimicrobial prescribing rates by dental surgeons are depicted in Figure 3 [51]. Table 1 describes when a particular antibiotic was introduced and first reported to have developed resistance in India [52-54].

**Table 1:** Introduction and first recorded resistance of selected antibiotics in India Source: [52-54] Table credit: Vishnu Desai

Antibiotic	Introduced in India	First resistance in India
Amoxicillin	1972	Early 2000s
Clindamycin	1968	Early 1990s
Azithromycin	1988	Early 2000s
Clarithromycin	1991	Early 2000s

Global AMR and the Importance of Surveillance Systems

The fundamental tenets of contemporary medicine and healthcare are at risk because of AMR’s complex and rapidly evolving problem [55-57]. AMR’s effects are concerning. According to estimates, HIV killed almost 1.3 million people globally in 2019. If nothing is done to address the problem, that figure could increase to 10 million deaths annually by 2050 [55,57]. AMR has led to a substantial increase in healthcare costs along with a rise in mortality rates. The United States spends over $4.6 billion yearly to address just six major AMR threats [57,58]. AMR has a considerable economic impact as well. Even under an optimistic low-AMR scenario, the World Bank Group projected that the world economy could lose about $1.1 trillion by 2030, possibly increasing to $2 trillion annually by 2050 [20,57,59]. The selection pressure brought about by the overuse and abuse of antibiotics accelerates the development of AMR [60]. The existence of an antimicrobial agent generates selective pressure that, from an evolutionary perspective, encourages the emergence of AMR. However, inappropriate use of antibiotics, such as abuse and overuse, critically increases this selective pressure and hastens the emergence of resistance [57,61]. The demand for antibiotics by consumers or patients, the availability and accessibility of these medications, knowledge of their proper use, insufficient disease diagnosis, and the availability of substandard antimicrobial drugs are some of the factors that have been identified as major contributors to the misuse and overuse of antimicrobials in human health [62]. Critically essential antimicrobials are primarily divided into the Watch and Reserve groups under WHO’s AWaRe classification system, which was created to assess and track antimicrobial use. These categories include antibiotics that are used as a last resort for treating human diseases (Reserve) and have a higher potential to foster bacterial resistance (Watch) [57]. Recent reports indicate a rise in resistance to these high-priority antibiotics [57,63,64].

With the rapid increase in AMR, tracking antimicrobial consumption (AMC) and AMR is crucial for identifying resistance patterns. Surveillance efforts are essential for shaping AMR stewardship programs and policies [65-67]. Much work has been done to monitor AMR and AMC at the national, regional, and international levels. One notable instance includes the European Antimicrobial Resistance Surveillance Network (EARS-Net), which was established by 29 countries and is the largest regional surveillance network in the world [68]. As the core of the Global Action Plan, WHO initiated its first worldwide cooperative effort to improve and control AMR surveillance in 2015. This effort led to the Global Antimicrobial Resistance and Use Surveillance System (GLASS), which aims to strengthen the evidence supporting AMR [69]. Tracking and disclosing AMR prevalence and changes in common bacteria was the main goal of the GLASS initiative’s first phase, which ran from 2015 to 2019.

By the first data call in 2017, 42 countries had enrolled; by 2020, this number had risen to 126 nations [70,71]. Concerningly high levels of AMR, particularly in bloodstream infections, were noted in WHO’s 2022 GLASS report. The third most frequent organism that causes these infections, *Klebsiella pneumoniae*, has grown more resistant to third-generation cephalosporins, it was mentioned. This has increased the need for carbapenems as a last option, which has helped carbapenem-resistant *Enterobacterales *proliferate throughout the world. Furthermore, *Acinetobacter *spp. exhibit concerning carbapenem and aminoglycoside resistance rates, which is concerning because carbapenem-resistant organisms are frequently MDR and associated with treatment failures [63,71,72]. The usage of antibiotics, which is impacted by many variables in different nations and areas, was strongly associated with these increased resistance levels [73].

AMR Transmission’s Impact on Humans and the Use of Antimicrobials in Food Animals

Antimicrobial usage in animals: Antimicrobials have been widely used to prevent, treat, and manage disease. In animal husbandry, they have also been essential growth promoters [74,75]. For over 50 years, the United States and other wealthy nations have utilized antimicrobial growth promoters (AGPs) in animal agriculture. Moore et al. (1946) and Stokstad and Jukes (1950) reported that supplementing sulfasuxidine and streptothricin antimicrobials with AGPs in pig and chicken feed enhanced animal food production [76,77]. They were initially introduced in the mid-1950s [76,77]. The US FDA authorized using AGPs in animals in 1951. According to current estimates, the average annual intake of antimicrobials per kilogram of animal product worldwide is 172 mg/kg for pigs, 148 mg/kg for chickens, and 45 mg/kg for cattle. Based on this foundation, global antibiotic usage is projected to increase by 67% between 2010 and 2030, rising from 63,151 ± 1,560 tons to 105,596 ± 3,605 tons. In the EU, antibiotics are now used to treat 8,927 tons of animals [78,79]. In the United States, food animals received about 14,600 tons of subtherapeutic antibiotic dosages in 2012 [69]. China, which produces and uses the most antimicrobials globally, employed 29,774.09 tons of antibacterial chemicals in animal husbandry in 2018. Of this amount, 53.20% was consumed to aid in the growth of the animals [80]. Viral infections are thought to have cost the aquaculture sectors in Malaysia, China, Mexico, Thailand, and Vietnam more than $44 billion between 2010 and 2016 [81].

Animals’ resistance to antibiotics and how it affects humans: Drug resistance brought on by the careless use of antibiotics endangers the health of both people and animals. Animal health is primarily impacted by antibiotic resistance in food animals, which can also result in resistant diseases in people. Following the administration of streptomycin to turkeys in 1951, the first documented instances of antibiotic resistance in food animals were reported [74,82-85]. Since then, there has been a rise in antibiotic resistance to tetracyclines, penicillin, and β-lactams [86]. A study on Escherichia coli samples from 198 cattle in eastern Algeria found that strains of the bacteria that produce extended-spectrum β-lactamases (ESBLs) and ampicillin and tetracycline resistance were highly prevalent [87]. According to a study on K. pneumoniae, two or three ESBL-producing genes (blaCTX-M, blaSHV, and blaTEM) were found in six strains isolated from patients and the environment [88]. MCR-1 was found in E. coli cultures from raw meat, animals, and individuals afflicted with E. coli between 2011 and 2014 [89]. Every year, antimicrobial-resistant microbes cause almost two million infections, over 23,000 deaths in the United States, and 25,000 fatalities in Europe [90,91]. AMR has led to ineffective treatments in 195,763 cases of pneumococcal disease and 2,925 child deaths in Ethiopia per year, with a 29.4% risk of failure for initial therapies [92].

AMR Prevention Techniques

**Figure 4:**
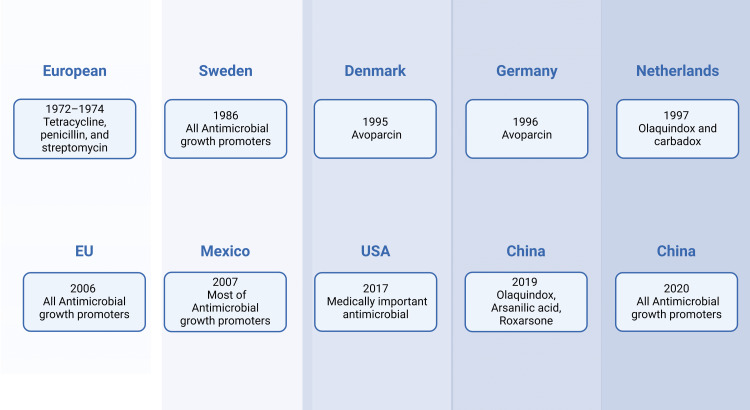
Overview of the legal prohibition of AGPs in producing food animals AGP, antimicrobial growth promoter This figure was created using the premium version of BioRender (biorender.com) under agreement license number NS281OXZ7. Illustration credit: Vishnu Desai

Many countries restrict or outright forbid the administration of antimicrobial agents as growth boosters in food animals (Figure 4). The health, productivity, and welfare of animals, as well as the potential for increased food costs, could all be significantly impacted by a complete and absolute prohibition on the use of antibiotics in food animals [93]. However, improvements in feed management and hygiene have been demonstrated to lessen the detrimental effects of such prohibitions on the productivity and health of animals [94,95]. It is also critical to remember that the initial advantages of AGPs, such as feed efficiency and weight gain, have gradually faded [96,97].

FAO, WHO, and the International Society for the Health of Animals are working together to control the use of antibiotics in animals [98]. The 2011 World Health Day theme, “Combating Drug Resistance: No Action Today, No Cure Tomorrow,” aimed to persuade nations worldwide to combat bacterial resistance more vigorously. A worldwide action plan to combat antibiotic resistance was also developed by the World Health Assembly in 2015. Furthermore, at the 2016 United Nations Higher Levels Conference for AMR, which took place at the G20 meeting in Hangzhou, foremost pledges to fight antibiotic resistance were made [99].

How to cut back on the use of antibiotics in primary care: In general practice, about 70% of antibiotic prescriptions are written, and research shows that at least 20% of these are inappropriate [100]. An estimated 4.95 million fatalities in 2019 were attributed to drug-resistant illnesses. Antibiotic-resistant illnesses and the deaths they cause are becoming more common [100].

Prescribing fewer antibiotics: Antibiotic resistance declines when antibiotic use declines. The well-known Finnish study on Streptococcus pyogenes, which is resistant to macrolides, showed how AMR could be reduced by consuming fewer macrolides. Between 1997 and 2000, the resistance rate dropped from 9.2% to 7.4%, demonstrating a statistically substantial association between antibiotic consumption levels and regional macrolide resistance [14]. To reduce antibiotic usage and promote appropriate prescribing practices, we only prescribe antibiotics to patients most likely to benefit [14]. Van der Velden and associates recently assessed how physician-targeted interventions affected primary care physicians’ ability to prescribe antibiotics more effectively for respiratory tract infections. A total of 58 studies were included in the analysis, which showed an 11.6% decrease in the prescription of antibiotics. Among the 59 interventions aimed at lowering the usage of antibiotics, concurrent interventions - which combined many strategies - proved to be more successful than single interventions that addressed a single problem. The most effective methods were multifaceted therapies with physician education materials [14]. Nevertheless, conflicting findings have been reported by other reviews. Generally speaking, a 10% increase in the prescription of first-choice antibiotics was associated with multiple interventions [14].

Government regulations that forbid the selling of antibiotics over the counter: In many parts of the world, self-medication with antibiotics is common. Antibiotics are unlawfully sold without a prescription in several nations. This mainly affects many nations in Asia, Africa, South and Central America, and even Southern European countries like Italy, Spain, Greece, and Malta [14].

Encouraging the application of reliable point-of-care assessments: The availability of diagnostic tools in Scandinavia is the fundamental distinction between a primary-care consultation in Scandinavia and one in a country in Southern Europe. Agar plates for urine culture and bacterial susceptibility testing (like Flexicult plates), CRP equipment to rule out severe respiratory infections, leukocyte analysis tools, and rapid antigen detection examinations for streptococcal pharyngitis are commonly available to GPs in the northern regions countries such as Denmark, Finland and Sweden. These diagnostic tools provide crucial information, enabling physicians to identify the bacterial cause of UTIs and assess microorganism susceptibility patterns within a day [22]. Point-of-care diagnostics provide the primary advantage of lowering physicians' uncertainty by providing insightful information that helps them determine whether patients should or shouldn't receive antibiotic treatment. Not all rapid tests, however, are appropriate for primary care; only those that are precise, accurate, quick, inexpensive, and simple to use and interpret are deemed appropriate in a primary care context [14].

Resistance to Antimicrobials in Developing Nations

Gu et al. (2021) reported that the global population continues to grow, reaching 7.8 billion by mid-2020, rising from 7 billion in 2010, 6 billion in 1998, and 5 billion in 1986 [101]. The International Monetary Fund determined that there are 152 developing countries with a population of around 6.90 billion (85.59%) [102] and responsible for 90% of global mortality [103]. The leading cause of the high prevalence of AMR in many LMICs is the lack of accurate and sufficient data on surveillance systems in these nations. Medical practitioners frequently prescribe broad-spectrum antibiotics, which can result in more serious resistance issues and allow the infection to destroy the patient’s microbiome [104,105]. Any AMR containment strategy must include surveillance since it offers the information required to identify an AMR issue. There are many obstacles to building surveillance systems, particularly in poor nations, such as the requirement for a considerable upfront expenditure [106,107]. Overcrowding and inadequate sanitization are two leading causes of the spread of antibiotic-resistant bacteria. Human-to-human contact with infected food, water, or other carriers can spread resistant microorganisms by contacting surfaces or items [108]. Establishing sufficient institutional, regulatory, and legal networks and frameworks [109]. Some difficulties include a lack of regulation surrounding the use of antibiotics in humans and animals, a lack of monitoring of antibiotic use and resistance levels, a lack of updated antimicrobial treatment protocols, and a lack of updated antimicrobial treatment guidelines [110]. Vaccines are the primary method of preventing infectious diseases. A person becomes protected against pathogens after vaccination, and the severity of the sickness also decreases [111].

Particular Dental Contributions and the Relationship Between Dentistry and the Use of Antibiotics Worldwide

About 10% of all prescriptions are written for dentistry, which adds considerably to the world's antibiotic consumption. Inappropriate use of antibiotics in dental treatment also leads to antibiotic resistance, a global health concern [112]. The overuse and abuse of antibiotics in dentistry may worsen because dental patients fail to follow their prescribed antimicrobial treatment [113]. Antibiotics are commonly used in dentistry to prevent and cure infections, particularly in cases of medication-related osteonecrosis of the jaw, pulp and preapical tissue disorders, odontogenic abscesses, and chronic apical periodontitis [114]. Regretfully, 80% of antibiotics used to treat acute dental conditions were unnecessary, according to a British study [115]. Thus, the use of antibiotics by doctors and dentists has been steadily rising, which has resulted in their abuse and overuse. Unfortunately, this has increased microbial resistance to antimicrobial agents, raising mortality rates, prolonging hospital stays, and decreasing patient protection against infectious diseases [116-118]. Dentists’ first-choice antimicrobials vary from nation to nation. Similar to the proportion of other antibiotics in our survey, co-amoxiclav is the preferred option in Spain [119,120], Turkey [121], and Serbia [122]. Studies conducted in Spain have also shown variations; the most recent study indicates an improved ratio of amoxicillin to co-amoxiclav. The dentists’ age determined the first-choice antibiotic in Serbia; younger dentists prescribed amoxicillin, while older dentists prescribed co-amoxiclav [122]. In dental treatment, antibiotics are also frequently prescribed for immunocompromised patients, patients exhibiting clear evidence of a systemic infection, and patients whose infection symptoms worsen quickly [122,123]. Systemic antimicrobials should only be used in acute periodontal disorders where drainage or debridement is not feasible, the infection has spread locally, or systemic dissemination has occurred. Antibiotics are not necessary for chronic inflammatory periodontal conditions [124]. The fact that between 2000 and 2015, the DDDs of antibiotics consumed in 76 countries increased by 65% (21.1-34.8 billion DDDs), and the rate of antibiotic consumption increased by 39% (11.3-15.7 DDDs per 1000 population per day), is evidence of the ineffectiveness of interventions around the world [125]. In this scenario, it has been recognized that it is necessary to control AMR by promoting coordinated interventions in different areas (Table 2) [126-129].

**Table 2:** Coordinated interventions to control AMR in dentistry AMR, antimicrobial resistance Source: [126-129] Table credit: Vishnu Desai

Serial no.	Coordinated interventions to control AMR in dentistry
1	Prescribe antibiotics only when necessary, following guidelines
2	Prevent the outbreak of infections by following strict hygiene rules, such as thorough handwashing and the use of personal protective equipment
3	Increase research and development of new vaccines and antibiotics
4	Appropriately manage environmental factors that promote AMR development and spread
5	Prescribe antibiotics with a lower potential for resistance development as empiric treatment’s first or second choice
6	Educate patients on the proper use of antibiotics and the problems associated with misuse to improve adherence
7	Improve surveillance of infections caused by resistant bacteria
8	Use vaccines that help limit the spread of AMR by preventing infections from occurring

Future research perspectives

Future research should concentrate on creating novel antimicrobial medicines and complementary therapies to treat drug-resistant illnesses. Better surveillance methods are required to monitor AMR patterns worldwide, particularly in LMICs. Research should examine how well ASPs work in various healthcare environments and evaluate the effects of more stringent antibiotic laws. Antibiotic usage can also be decreased via studies on the role of quick diagnostic techniques, vaccine development, and microbiome manipulation. A multidisciplinary strategy integrating environmental, animal, and human health is necessary for long-term AMR mitigation.

Limitations

The fact that this review is predicated on previously published works may limit the inclusion of recent or unpublished research and induce publication bias. Since the study primarily uses secondary data, it might not accurately reflect current developments in AMR, especially in LMIC nations with inadequate surveillance. Furthermore, the generalizability of results may be impacted by geographical variances in antibiotic use, resistance patterns, and disparities in study techniques. The review does not include primary data collection and clinical trials, which could offer more direct insights into the efficacy of interventions and ASPs.

Key features

**Figure 5:**
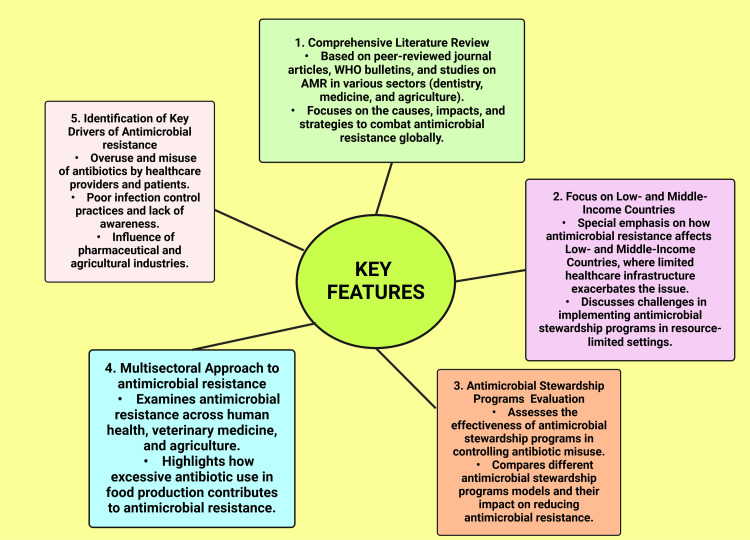
Key features of the narrative review This figure was created using the premium version of BioRender (biorender.com) under agreement license number KG281P71KO. Illustration credit: Vishnu Desai

Through an analysis of peer-reviewed literature, WHO publications, and studies on the use of antibiotics in agriculture and healthcare, this review study investigates AMR. It draws attention to the impact of AMR, especially in LMICs, where the problem is made worse by a lack of adequate healthcare infrastructure. The effectiveness of ASPs in reducing antibiotic abuse is compared in this research. A multisectoral approach analyzes AMR in agriculture, veterinary medicine, and human health while highlighting essential causes such as excessive antibiotic usage, inadequate infection control, and industry influence. Lastly, it offers evidence-based suggestions for lowering antibiotic abuse and bolstering international AMR response initiatives (Figure 5).

## Conclusions

According to our research, many dentists continue to prescribe antibiotics arbitrarily, often without a solid scientific foundation, depending instead on their own experience or antiquated procedures. The growing issue of antibiotic resistance is caused mainly by the imprudent use of antibiotics, especially when they are not therapeutically required. The reduction of needless antibiotic prescriptions depends on ongoing education and awareness campaigns that stress the significance of appropriate antibiotic management and rigorous adherence to biosafety procedures. One of the most critical tactics in the battle against AMR is antimicrobial stewardship. It entails a methodical strategy that promotes and supports the prudent use of antibiotics on a personal, governmental, and international scale. Antimicrobial stewardship also covers several topics, such as environmental concerns, animal health, and human health. The inappropriate use and misuse of antibiotics in animals and human medicine exacerbate AMR, one of the most pressing global health issues. The rise in multidrug-resistant diseases highlights the urgent need to enhance antibiotic stewardship, particularly in health care, dentistry, and agriculture. Although ASPs have been implemented more successfully, more work is needed to reduce the overprescription of antibiotics and raise awareness among the common people and healthcare professionals. Stricter laws governing antibiotics, better diagnostic equipment, and international collaboration are all necessary to combat AMR, especially in LMIC nations where the disease is most common. A more prudent approach to antibiotic use is required to reduce the growth of resistant infections and guarantee that necessary antibiotics are effective for future generations. Sustained efforts are needed to ensure proper antibiotic access, minimize abuse, and encourage research into alternative medicines to combat AMR effectively. A future in which common diseases become incurable and cause substantial morbidity and mortality can only be avoided by international cooperation and dedication to the responsible use of antibiotics.
